# Controlling surface wetting in high-alkaline electrolytes for single facet Pt oxygen evolution electrocatalytic activity mapping by scanning electrochemical cell microscopy†

**DOI:** 10.1039/d4sc04407j

**Published:** 2024-09-11

**Authors:** Geovane Arruda de Oliveira, Moonjoo Kim, Carla Santana Santos, Ndrina Limani, Taek Dong Chung, Emmanuel Batsa Tetteh, Wolfgang Schuhmann

**Affiliations:** a Analytical Chemistry – Center for Electrochemical Sciences (CES), Faculty of Chemistry and Biochemistry, Ruhr University Bochum, Universitätsstraße 150 D-44780 Bochum Germany wolfgang.schuhmann@rub.de; b Department of Chemistry, Seoul National University Seoul 08826 Republic of Korea; c Advanced Institutes of Convergence Technology Suwon-si 16229 Gyeonggi-do Republic of Korea

## Abstract

Scanning electrochemical cell microscopy (SECCM) has been used to explore structure-electrocatalytic activity relationships through high-resolution mapping of local activities of electrocatalysts. However, utilizing SECCM in strongly alkaline conditions presents a significant challenge due to the high wettability of the alkaline electrolyte leading to a substantial instability of the droplet in contact with the sample surface, and hence to unpredictable wetting and spreading of the electrolyte. The spreading phenomena in SECCM is confirmed by the electrochemical response of a free-diffusing redox probe and finite element method (FEM) simulations. Considering the significance of alkaline electrolytes in electrocatalysis, these wetting issues restrict the application of SECCM for electrocatalyst elucidation in highly alkaline electrolytes. We resolve this issue by incorporating a small percentage of polyvinylpyrrolidone (PVP) in the electrolyte inside the SECCM capillary to increase the surface tension of the electrolyte. To demonstrate successful wetting mitigation and stable SECCM mapping, we performed oxygen evolution reaction (OER) mapping on polycrystalline Pt by using 1 M KOH with an optimized PVP concentration. The OER activity maps correlated with the orientation of the exposed facets determined by electron backscatter diffraction and reveal different activities between Pt facets, hence confirming our methodology for exploring electrocatalytic activities in single facet scale in concentrated alkaline media. Interestingly, the maximum OER current density was highest for (110) and (111) which contradicts the activity trends in acidic electrolyte for which (100) is most active for the OER.

## Abstract 2

SECCM experiments in concentrated alkaline solutions are challenging due to the surface wetting. We propose a stable SECCM method which enables resolving high-activity sites for electrocatalysis at high pH (*e.g.*, 1 M KOH).

## Graphical abstract

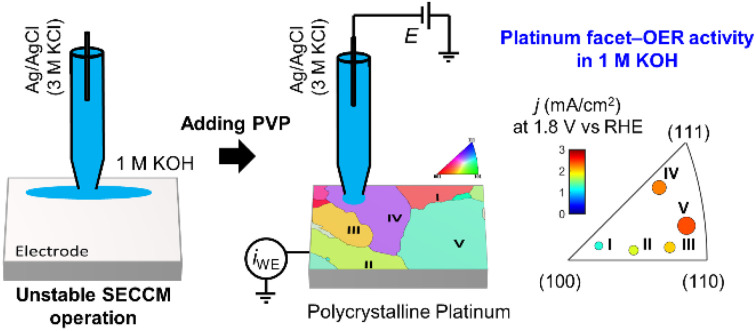

## Introduction

Scanning electrochemical cell microscopy (SECCM) is powerful for identifying local electrocatalytic activities at the nano/micrometer scale,^1^*e.g.* single facets,^2^ and single nanoparticles.^3,4^ Correlative analysis combining SECCM with other microscopy techniques such as electron backscatter diffraction (EBSD) and atomic force microscopy (AFM) enables understanding local activities related to local crystal orientation and morphology. The fast mass transport in the SECCM configuration enables efficient evaluation of intrinsic electrocatalytic activities due to the nano/micrometer sized electrode area, fostering rapid diffusion of species through hemispherical-like diffusion.^5^ Moreover, the electrode–electrolyte droplet-gas geometry, which mimics the 3-phase boundary of gas-diffusion electrodes (GDE), facilitates the supply of reactant gas and the removal of product gas from the surface.^6^ This allows the interrogation of intrinsic kinetics at high current density for electrocatalytic reaction involving gas evolution or gas consumption.

The spatial resolution of SECCM is determined by the size of the droplet formed on the surface of the SECCM tip. It is mainly influenced by the tip diameter, but the properties of the electrolyte, like viscosity and the surface tension, also have significant effects. Thus, there are limitations for stable SECCM operation. For instance, while SECCM has been widely used to map the electrochemical activity of a variety of materials in neutral or acidic conditions,^2,7^ there have been few applications of alkaline electrolytes.^8^ This scarcity of studies can be attributed to the challenge of maintaining consistent droplet sizes in highly concentrated alkaline electrolytes.^9^ Under such conditions, the low surface tension of the electrolyte can lead to spreading phenomena after landing on the surface, complicating long-term experiments and high spatial resolution analyses.^8^ So far, to obtain electrocatalytic activity mapping data in alkaline electrolyte, the concentration of the used KOH was intentionally lowered to 0.01 M (ref. 10 and 11) and/or the wall of the pipette was silanized to make it hydrophobic (0.01 and 0.05 M KOH).^8,10,12^ However, in high concentration alkaline electrolytes, silanization alone cannot prevent spreading of the electrolyte, causing a pancake like-shaped spread droplet with a large and unstable wetted surface area.^8^ It has also been shown that 0.1 M NaOH can be used in SECCM measurements without significant wetting, but only with highly-ordered pyrolytic graphite (HOPG) substrate for the borohydride oxidation reaction (BOR).^13^ Similarly, the usage of highly concentrated KOH, such as 0.1 M KOH, has been made possible using thin-layer oil-covered electrodes.^4,14^ However, this method has practical challenges; it can obscure the location of the tip during the approach, potentially complicating its visualization. It also might disable one of the main advantages of the SECCM, namely the presence of a 3-phase boundary, which is essential for studying fast kinetics of the reactions involving gaseous reactants. Another way for stabilizing SECCM droplets involves gelation of the electrolyte incorporating polyacrylamide (PAM) gel.^9^ The SECCM tip is filled with a monomer solution mixed with the supporting electrolyte, KCl, followed by gelation with UV light. Despite enabling high spatial resolution imaging of live cells and electrodes,^9,15^ this technique cannot be applied at high alkaline conditions due to the hydrolysis of PAM in such environments.^16^ Moreover, high concentrations of polymers modify the solvation properties of the electrolytes, which may affect electrocatalytic activities. Considering the importance of alkaline conditions in electrocatalysis,^17^ a feasible strategy to mitigate alkaline droplet spreading while not disturbing the chemical nature of the electrolytes is in demand. The stable use of SECCM in strong alkaline conditions will make it possible to investigate transition metal-based electrocatalysts which cannot be applied in acidic conditions.

In this work, we incorporate a small percentage of a redox-inactive polymer, polyvinylpyrrolidone (PVP) in 1 M KOH, into the electrolyte filled into the tip capillary for stable and robust electrocatalytic SECCM mapping in highly alkaline conditions. PVP was chosen because of its high resistance against alkaline electrolytes and corrosion.^18,19^ PVP is a versatile polymer that has been widely used in various fields due to its chemical stability and ability to improve material properties. It finds applications in areas such as corrosion resistance,^20^ pharmaceutics,^21^ biosensors^22^ and polymer electrolyte membrane fuel cells (PEMFCs).^19,23^ The spreading behavior in 1 M KOH was experimentally monitored by sequentially recording voltammograms of a reversible redox species in absence and presence PVP, and confirmed by finite element method (FEM) simulation. Scanning electron microscopy (SEM) images of the trace of electrolytes after SECCM experiments demonstrate the decreased wetted area. The optimized conditions were applied to SECCM mapping of OER at a Pt electrode with large grain boundaries at pH 13.8. The correlation of the SECCM result to the EBSD map provides the Pt grain orientation-dependent OER activity in highly alkaline electrolyte, which surprisingly differs from previous findings obtained in acidic electrolytes.

## Results and discussion

### Experimental investigation of the wetting behavior in alkaline electrolytes using a redox mediator

**Fig. 1:**
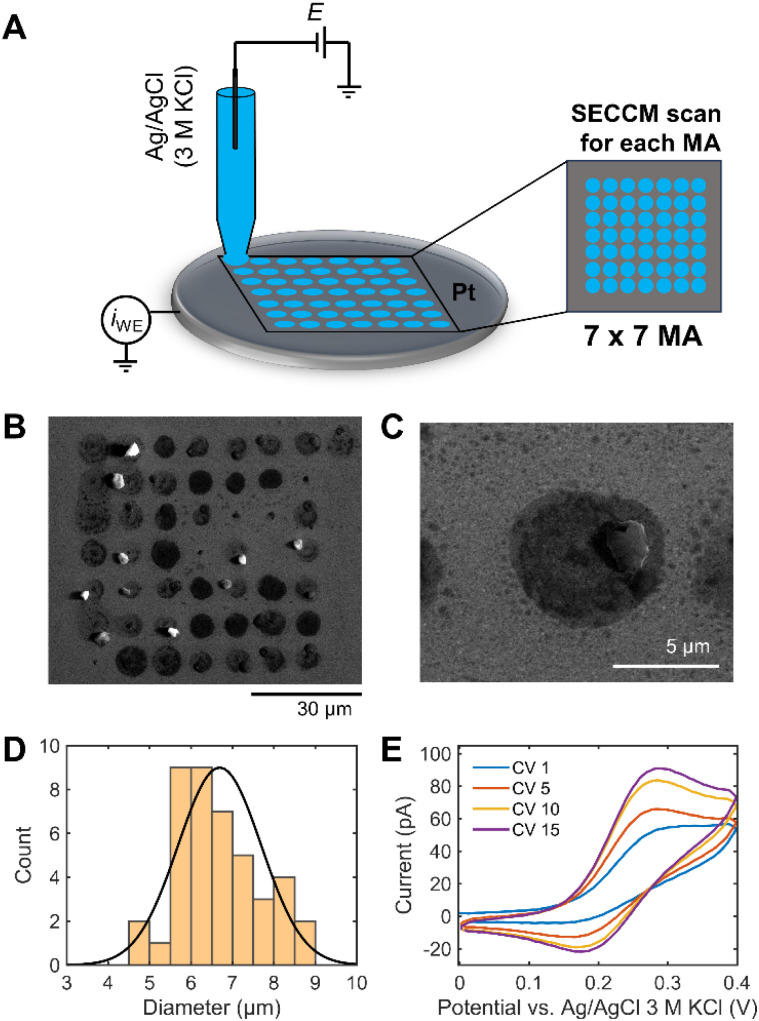
(A) Schematic illustration of the SECCM configuration in hopping mode with 7 × 7 spots per measurement area (MA). (B) SEM image of the 49 landing spots. (C) Zoomed SEM image of a single spot showing crystallized KOH debris remaining on the Pt surface. (D) Size distribution of the area of 42 landing spots with a normal distribution fit (6.7 ± 1.0 μm). (E) Sequence of 15 CVs at 1 V s^−1^ for 1 M KOH + 1 mM Fc(MeOH)_2_ recorded at a Pt surface of a single SECCM tip landing site.

A 7 × 7 scan SECCM hopping experiment in 1 M KOH containing 1 mM ferrocene dimethanol (Fc(MeOH)_2_) on a Pt electrode was performed to demonstrate the challenge of reliable SECCM operation in strong alkaline conditions (Fig. 1A). It is worth to note that quartz capillaries were used for the SECCM tips for alkaline electrolytes to decrease etching of the pipettes by the high-alkaline electrolyte. Quartz has a 10–1000 times lower Si dissolution rate than other types of silicates.^24^ Fc(MeOH)_2_ is a pH-independent redox probe and is used to electrochemically monitor the wetting behavior of the droplet during the SECCM experiment. A SECCM tip with *ca.* 900 nm diameter comprising a silanized glass pipette for confining electrolyte wetting exclusively to the sample surface was used (Fig. S1†). Fig. 1B shows the SEM image of 49 landing sites after SECCM scanning on a planar Pt surface. After droplet landing, 5 consecutive cyclic voltammograms (CV) were performed within the designated measurement area (MA). The solution spreads over a larger area than the capillary size upon landing on the Pt surface. Additionally, water evaporation from the electrolyte droplet increases the pH, resulting in a visible white/gray crystallized residue after the experiment (Fig. 1C).

Alkaline electrolytes exhibit lower contact angles on a Pt surface than acidic electrolytes (Fig. S2†). The interaction between adsorption of OH^−^ and water molecules can make the surface hydrophilic, leading to the low surface tension of the substrate–liquid interface.^25^ The decreasing contact angle of the KOH solution with increasing concentration can facilitate the solution flowing out of the capillary tip with increasing droplet area. The size of each landing spot was derived, revealing a diameter of the wetted area of approximately 7 times larger than the capillary size (Fig. 1D).

We define a wetting factor (WF), representing the degree of wetting (WF = the spreading droplet diameter/the tip end diameter), which is 7 for this case. The significant standard deviation of the wetted area indicates poorly controlled conditions for investigating electrochemical responses in 1 M KOH using SECCM. Fig. 1E shows a sequence of CVs of Fc(MeOH)_2_ performed consecutively at the Pt surface of a single tip landing site. Fc(MeOH)_2_ undergoes fast outer-sphere electron transfer and hence the CV is influenced by the mass transport which is in turn influenced by the shape of the droplet in contact with the electrode surface. The current increases during the consecutive CVs and the shape of the redox waves changes from more sigmoidal to more peak-shaped indicating spreading of the droplet on the surface with time. The peak-shaped CV is characteristic of a thin-layer cell electrochemistry originating from the pancake-shaped spread droplet geometry, as reported previously by Varhade *et al.*^8^ The impact of droplet spreading on the shape of the CV will be discussed in detail using FEM simulation below.

We repeated the experiment using K_3_[Fe(CN)_6_] as alternative redox probe to verify that the redox probe does not impact on the droplet meniscus stability (Fig. S3A†). The sequence of CVs displayed the increasing redox peak current, indicating that the wetted area was expanding with time to reach a WF of 5. The findings, depicted in Fig. S3† resemble those obtained with Fc(MeOH)_2_. Additionally, the same experiment was repeated in the absence of a redox probe, *i.e.*, in 1 M KOH. The CVs show increasing capacitive currents, which can be attributed to the larger wetting-area also seen in the SEM images (Fig. S4†). These results confirm the spreading effect independently of a free-diffusion redox species in 1 M KOH, implying that, as expected, the concentrated alkaline electrolyte is the dominant factor for surface wetting.

### Electrochemical behavior of the SECCM response in spreading droplets

**Fig. 2:**
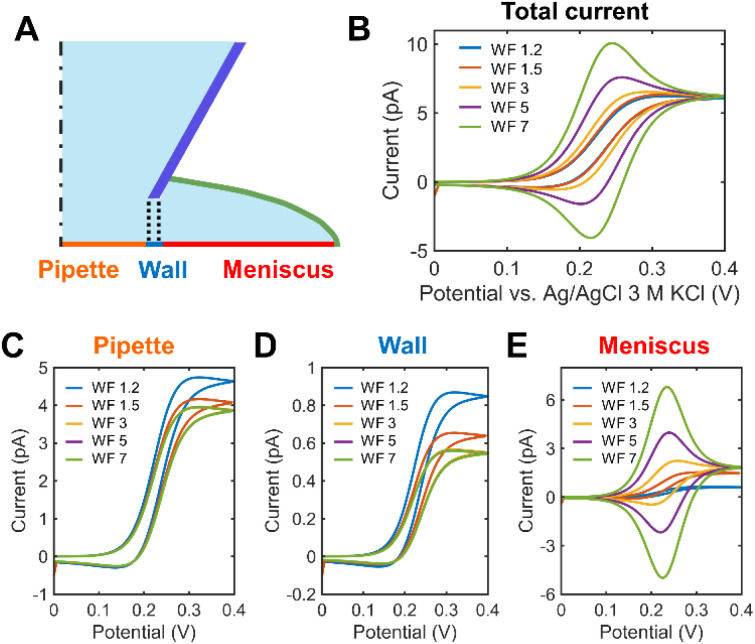
SECCM voltammograms depending on the droplet diameter (*d*_drp_) while the tip diameter (*d*_tip_) is fixed to 900 nm. (A) The electrode surface was divided into three areas according to the types of the dominant mass transport flux. (B) Overall voltammogram of 1 mM Fc(MeOH)_2_ with a scan rate of 1 V s^−1^ in dependence of the wetting factor (WF). (C–E) The contribution of (C) pipette, (D) wall, and (E) meniscus parts to the overall voltammogram.

FEM simulation was performed to systematically explore the effects of the droplet spreading on the SECCM voltammograms in parameterized SECCM tip-droplet geometries including the WF, the semi-angle of the tip (*θ*), and the droplet thickness (*h*) (Fig. S5†). Fig. 2 demonstrates the effects of droplet spreading on the SECCM voltammograms in 1 mM Fc(MeOH)_2_ at various WF values. As depicted in Fig. 2B, the droplet spreading changes the voltammogram shape from sigmoidal to peak-shaped, caused by the pancake-shaped droplet at alkaline conditions. It adds the thin-layer cell electrochemical nature to the voltammogram.^8^Fig. 2C–E compares the contribution of each electrode surface. The pipette surface, facing the direct mass transport flux from the tip, exhibits a sigmoidal current fixed regardless of the WF, attributed to the spherical diffusion along the tip (Fig. 2C). In contrast, the peak-shaped voltammogram at the meniscus surface is significantly affected by the WF. This phenomenon arises from the spreading of the droplet, effectively acting as a thin-layer cell, which induces the surface-confined mass transport. In other words, the SECCM voltammogram is the result of the spherical diffusion with a limited semi-angle along the tip and the thin-layer cell diffusion confined to the droplet (Fig. S6†). This implies that it is necessary to consider the droplet spreading as well for an explicit understanding of the SECCM electrochemistry.

**Fig. 3:**
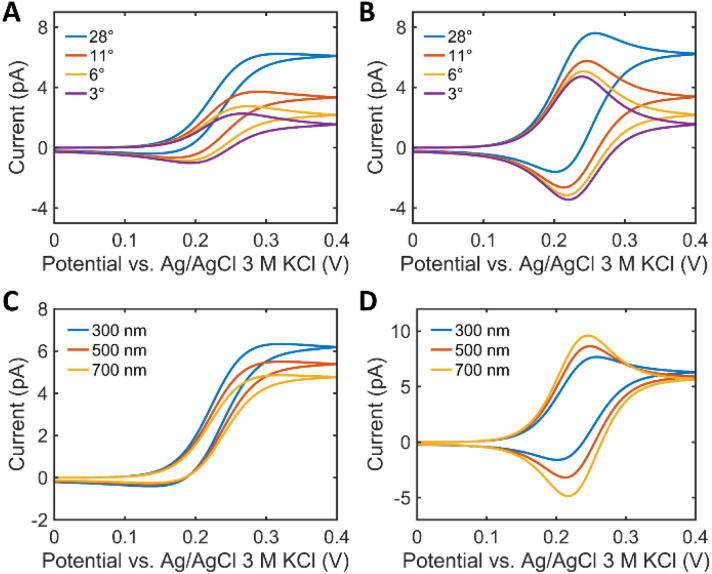
The effect of the tip slope and the droplet thickness on the voltammograms. (A and B) SECCM voltammogram of 1 mM Fc(MeOH)_2_ with the semi-angle (*θ*) of 3°, 6°, 11°, and 28° at a WF of (A) 1.2 and (B) 5. (C and D) SECCM voltammogram of 1 mM Fc(MeOH)_2_ with the *θ* of 28° as a function of the droplet thickness (*h*) when the WF is (C) 1.2 and (D) 5.

Fig. 3A and B compares the voltammograms in dependence on the semi-angle *θ* of the tip. The current increases with *θ*, regardless of the WF because at a higher angle the wider opening promotes the spherical diffusion of the reactant along the tip. The shape of the voltammogram at WF 5 varies as a function of *θ*. It becomes more bell-shaped with decreasing *θ* (Fig. 3B). Fig. S7† summarizes the anodic peak current (*i*_anodic peak_), the limiting current (*i*_lim_) and the normalized *i*_lim_ according to the diameter of the spread droplet. Droplet spreading leads to a slight increase of normalized *i*_lim_ (Fig. S7C†), however, the changes are negligible (Fig. S7B†), as in previous studies.^26^ In contrast, the peak current depends on both *θ* and the WF (Fig. S7A†). Fig. 3C and D shows voltammograms as a function of *h* at different WF values. The current trend exhibits opposite behaviors depending on the WF values. When the WF is small, the major mass transport mechanism is the spherical diffusion along the droplet and the tip. The increase in *θ*, which means the wider opening of the passage for the flux, leads to the faster mass transport. Since the tip has a bigger *θ* than the droplet, the increase in *h* of the droplet results in a decrease in the overall spherical diffusional flux. On the other hand, when the WF is big, no notable increase in the overall current is observed. *i*_anodic peak_ becomes bigger with *h* since the volume of the thin layer cell grows, and hence, as in thin layer electrochemistry, the peak current is proportional to the cell volume.^27^

### The PVP concentration dependent wetting behavior

To mitigate the wetting effect, we aimed to control the wettability of the electrolyte by introducing a suitable polymer. The selected thickening agent should be redox-inactive and should have good ionic conductivity and high resistance to strong alkaline condition.

**Fig. 4:**
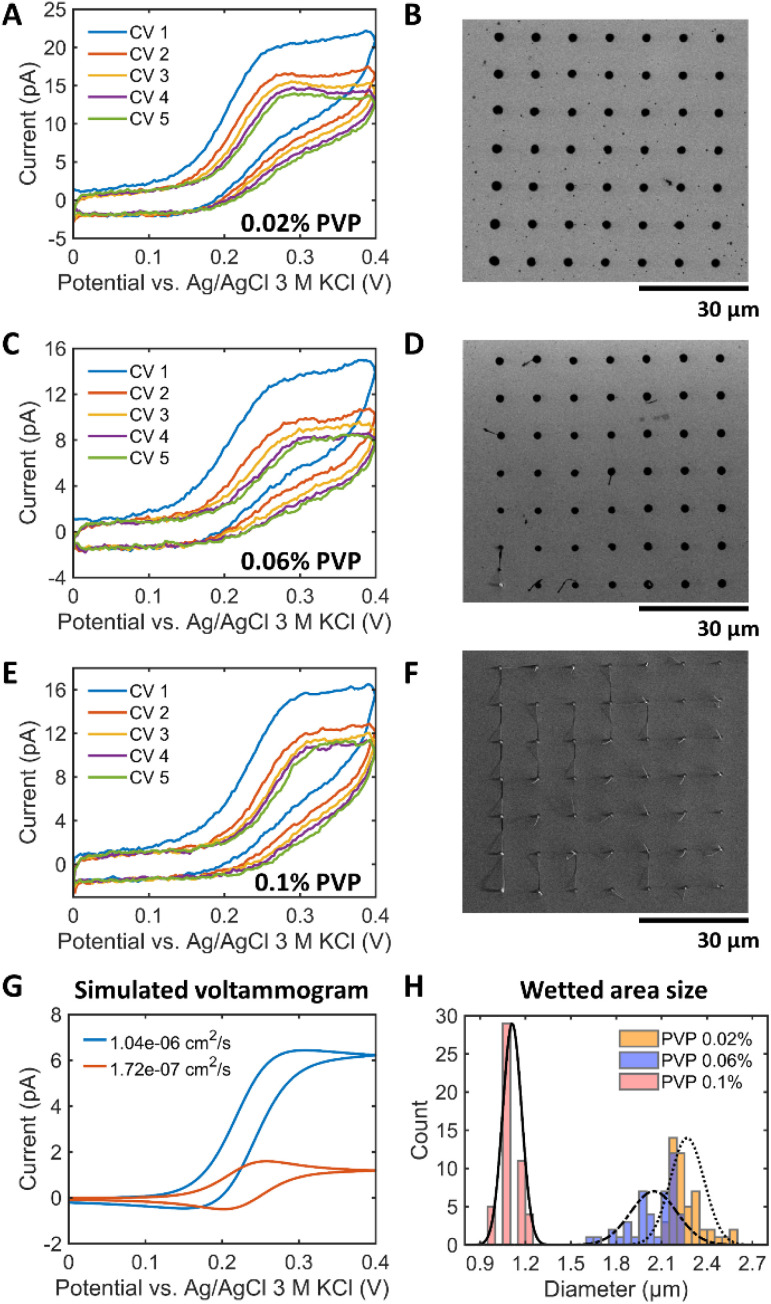
SECCM voltammogram and the size of landing spots in dependence of the PVP concentration. (A, C, E) Representative SECCM CVs (5 cycles) at 1 V s^−1^ in the presence of 1 mM Fc(MeOH)_2_ in 1 M KOH and PVP (A) 0.02%, (C) 0.06%, (E) 0.1%. (B, D, F) SEM image showing landing spots after the SECCM experiments for PVP concentrations of (B) 0.02%, (D) 0.06%, and (F) 0.1%. (G) Simulated SECCM voltammograms for 1 mM Fc(MeOH)_2_ at 1 V s^−1^ with the same tip-droplet geometry and the diffusion coefficient values of 1.04 10^−6^ cm^2^ s^−1^ (without PVP) and 1.72 10^−7^ cm^2^ s^−1^ (PVP 0.06%). (H) The size distribution of the landing spots measured in the SEM images (B, D, F) with normal distribution fits scaled to maximum (0.02% PVP: 2.27 ± 0.11 μm, 0.06% PVP: 2.05 ± 0.15 μm, and 0.1% PVP: 1.11 ± 0.06 μm).

First, we considered the polymers utilized in alkaline polymer electrolyte membrane fuel cells (PEMFCs), such as polyethylene glycol (PEG), polyvinyl alcohol (PVA), PVP, and methyl 2-hydroxyethyl cellulose (MHEC).^19,28^ Among them, amorphous polymers like PVP and MHEC exhibit a better ionic mobility than crystalline soluble polymers like PEG and PVA. The absence of a crystalline structure allows for greater mobility of ions within the material^19^ and thereby can reduce the solution resistance. However, MHEC was not suitable for the SECCM experiment because of a low current (Fig. S8A†) and a sticky polymer residue at the end of the tip (Fig. S8B†). Consequently, PVP was selected to tune the wettability of the electrolyte inside the SECCM capillary. Different PVP concentrations of 0.02%, 0.06% and 0.1% were introduced into the 1 M KOH solution. It should be noted that adding PVP did not change the pH of the KOH solution, which is pH 13.8 (Table S2†). The contact angles of the 1 M KOH solution increased with the PVP concentration (Fig. S9A†). Fig. 4A–F shows the results for a 900 nm capillary filled with 1 mM Fc(MeOH)_2_ + 1 M KOH + *X*% (w/v, *X* g of PVP per 100 mL solution) PVP (*X* = 0.02, 0.06, or 0.1) in a hopping mode SECCM scan on a Pt surface. In contrary to the observed trends illustrated in Fig. 1E, 5 successive voltammetric cycles at the same landing location did not exhibit an increase in peak current. Instead, the current slightly decreased and stabilized after the third cycle. Furthermore, in contrast to the peak-shaped voltammograms in the polymer-free electrolyte, a sigmoidal-type profile with lower currents was obtained in the presence of PVP. Fig. 4B, D and F display the reduced wetted area due to the increase of the surface tension. Experiments using PVP in acidic electrolyte were also performed using 1 M HClO_4_ + 0.06% PVP (Fig. S10†). The addition of PVP to the acidic electrolyte resulted in small and reproducible landing spots, demonstrating the general applicability of the proposed addition for both acidic and alkaline electrolytes. As summarized in Fig. 4H, 1 M KOH with 0.02%, 0.06% and 0.1% PVP exhibited a WF of *ca.* 2.5, 2.3 and 1.2 times, respectively. The decreased standard deviation of the wetted area with PVP indicates more controlled and reproducible landing of the SECCM tip on the surface (Fig. S11†), even if, *e.g.* the presence of 0.1% PVP, led to the formation of electrolyte tails as shown in Fig. 4F.

To understand the voltammetric changes upon addition of PVP, we measured the diffusion coefficient *D* of electrolytes with and without PVP and performed the corresponding FEM modeling. *D* was determined by applying Randles–Ševčík equation to the voltammograms on a Pt macroelectrode in a solution of 1 M KOH + 1 mM Fc(MeOH)_2_ + PVP. The results are shown in Fig. S12 and Table S3.† The solution with PVP has about 5 times lower *D* compared to solutions without polymer. Fig. 4G compares the simulated voltammograms with *D* values of Fc(MeOH)_2_ measured in 1 M KOH with and without 0.06% PVP with the same wetted area. It corroborates that the decrease in *D* of the redox mediator leads to a decrease in the limiting current proportional to *D* even though the tip and the droplet geometry do not change. This is attributed to the fact that the mass transport flux is proportional to *D*. This effect is visible in the voltammograms (Fig. 4A, C, and E) demonstrating a decrease in current by about 5 times compared to the polymer-free solution (Fig. 1E). Unlike the expectation that an increase in polymer concentration should lead to a decrease in *D* due to higher solution viscosity, raising the polymer concentration from 0.02% to 0.1% did not significantly alter *D*. In conclusion, PVP plays a vital role in managing droplet stability, confirming its effectiveness in preventing wetting problems in alkaline electrolytes. The stabilized droplet without spreading leads to sigmoidal voltammograms mainly controlled by the mass transport along the tip.

### Alkaline OER mapping at polycrystalline platinum surfaces

**Fig. 5:**
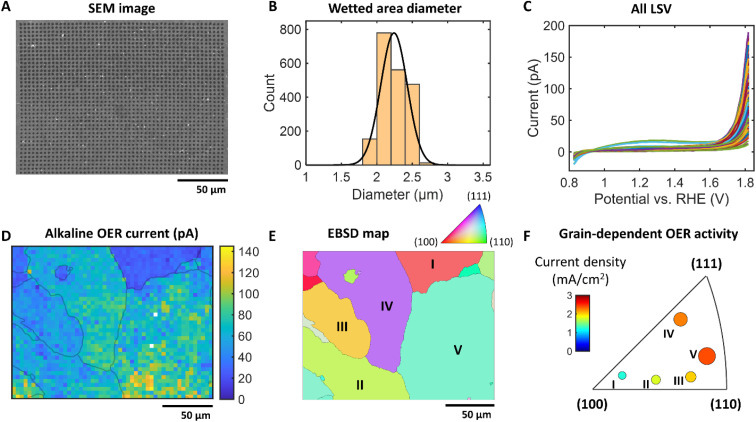
OER activity mapping of polycrystalline Pt by SECCM in 1 M KOH and 0.06% PVP (pH 13.8). (A) SEM image of the electrode after the SECCM experiment. (B) The size distribution of the landing spots with a normal distribution fit (2.24 ± 0.19 μm). (C) Mean linear sweep voltammograms (LSV) for each grain. Each shadow represents the standard deviation for each grain. (D) Current density map at 1.8 V *vs.* RHE overlaid with the grain boundaries from the EBSD data. (E) Normal direction (ND) crystallographic orientation map of the polycrystalline Pt where the SECCM experiment was conducted. (F) Electrochemical inverse pole figure (IPF) diagram showing the mean OER current density at 1.8 V *vs.* RHE of each grain. The size of each circle represents the relative size.

The OER activity of a Pt plate with large grain boundaries was investigated as a model reaction using a 770 nm silanized pipette filled with 1 M KOH + 0.06% PVP (pH 13.8) to demonstrate the applicability of the proposed methodology to prevent surface wetting. Upon each SECCM tip landing, a linear sweep voltammogram (LSV) was recorded in air and the OER was investigated within a defined area of 200 μm × 152 μm, with a hopping distance of 4 μm. The SEM image in Fig. 5A shows the reproducible and uniform landing spot sizes with a narrow size distribution (WF about 2.9; Fig. 5B). This represents a 58% reduction in the WF compared to conditions in the absence of polymer, highlighting the effectiveness of PVP in reducing wetting when using high alkaline electrolytes. Fig. 5C displays the LSVs measured in the SECCM experiment. The spatially resolved SECCM current map at 1.8 V *vs.* RHE in Fig. 5D reveals the heterogeneity of the OER activities. The overlay of the grain boundaries extracted from the EBSD data (Fig. 5E) to the current map confirms the Pt grain-dependent OER activities. Fig. 5E displays the normal direction (ND) orientation of the Pt grains. Among these grains, the analysis focused on the five largest ones, labeled *I*–*V*, for a detailed investigation. The averaged current values at 1.8 V *vs.* RHE of each grain are plotted in the inverse pole figure (IPF) (Fig. 5F). Ordering the maximum OER current density from (110) and (111) to (100) displays a decrease in current density. Interestingly, this is a different trend in comparison to the literature that shows an activity trend measured in HClO_4_ ^29^ and H_2_SO_4_ solution^30^ which is higher for (100) than for (110) and (111). This implies that the optimum Pt structures for high catalytic OER activities depend on the pH of the electrolyte, and therefore the design of OER catalyst active sites should be tailored with respect to the anticipated pH value during the electrocatalytic reaction. Notably, the SECCM measurement of reactions using strong alkaline electrolytes has not been feasible until now due to the spreading factor mitigated in this study. This underscores the significance of our findings in expanding the understanding of electrochemical behavior under alkaline conditions.

## Conclusions

In this study, the problematic unstable SECCM operation in concentrated alkaline electrolyte investigated and a solution is proposed. More comprehensive insights about the droplet spreading effects on voltammograms were experimentally revealed using a free-diffusion redox probe and further studied *via* numerical FEM simulations. We mitigate the wetting effect by incorporating a small amount of PVP into the alkaline electrolyte inside the SECCM capillary. PVP controls the wettability of the electrolyte and enhances the droplet stability in concentrated alkaline electrolytes. After optimization concerning the PVP amount supported by FEM simulations the proposed strategy was used to investigate the exposed facet-depending OER activity of polycrystalline Pt with large grains. SECCM measurement of a polycrystalline Pt surface combined with EBSD using high alkaline electrolytes at pH 13.8 unveils insights into the correlation between OER activity and the crystallographic orientation of grains on the polycrystalline Pt surface. The OER activity trend among the grains was similar for (110) and (111) orientations, both higher than for (100). Our robust activity mapping strategy with PVP has general applications for identifying high activity structures of electrocatalysts for alkaline electrocatalysis.

## Data availability

Data for this article are available at Zenodo at https://www.10.5281/zenodo.13499338. The data will be made open available after acceptance of the manuscript in Chem. Sci.

## Author contributions

W. S. and G. A. O. conceived the initial idea. G. A. O., M. K., E. B. T., and W. S. planned the experiments. G. A. O. conducted SECCM experiments and analysed the data. M. K. conducted the COMSOL simulation and EBSD analysis. N. L. participated in initial investigations of the polymers. G. A. O. and M. K. prepared the initial draft of the manuscript. T. D. C. offered COMSOL and suggestions for its analysis. C. S. S. provided constructive suggestions for the conceptualization and writing. W. S. supervised the study. All authors participated in revising the manuscript and have given approval to the final version of the manuscript.

## Conflicts of interest

There are no conflicts to declare.

## Supplementary Material

SC-OLF-D4SC04407J-s001
