# Supplementary material for: Controlling surface wetting in high-alkaline electrolytes for single facet Pt oxygen evolution electrocatalytic activity mapping by scanning electrochemical cell microscopy
Source: Chem Sci. 2024 Sep 11;15(39):16331–7. doi: 10.1039/d4sc04407j (PMC11409436; doi:10.1039/d4sc04407j)
Supplement: SC-015-D4SC04407J-s001 [file SC-015-D4SC04407J-s001.pdf]

Supplementary Information (SI) for Chemical Science.
This journal is © The Royal Society of Chemistry 2024

SUPPORTING INFORMATION

Controlling Surface Wetting in High-Alkaline Electrolytes for Single Facet Pt Oxygen Evolution Electrocatalytic Activity Mapping by Scanning Electrochemical Cell Microscopy

Geovane Arruda de Oliveira,[a†] Moonjoo Kim,[a†] Carla Santana Santos,[a] Ndrina Limani,[a] Taek Dong Chung,[b,c] Emmanuel Batsa Tetteh,[a] Wolfgang Schuhmann[a*]

[a.] *Analytical Chemistry – Center for Electrochemical Sciences (CES), Faculty of Chemistry and Biochemistry, Ruhr University Bochum, Universitätsstr. 150, Bochum D-44780, Germany*

[b.] *Department of Chemistry; Seoul National University; Seoul 08826, Republic of Korea*

[c.] *Advanced Institutes of Convergence Technology, Suwon-si 16229, Gyeonggi-do, Republic of Korea*

Correspondence to: wolfgang.schuhmann@rub.de

Material and methods

Chemicals

Potassium hydroxide (KOH) was purchased from Fisher chemical. 1,1'-ferrocenedimethanol ($Fc(MeOH)_2$) was purchased from ThermoFisher. Hexacyanoferrate(III) ($K_3[Fe(CN)_6]]$, dichlorodimethylsilane ($SiCl_2(CH_3)_2$), methyl 2-hydroxyethyl cellulose (MHEC) and polyvinylpyrrolidone (PVP) were purchased from Sigma-Aldrich. The average molecular weight of the PVP is 55,000, and the K value is 28–34. The K value represents the viscosity of the polymer solution. Consequently, PVP with a higher K value exhibits higher solution viscosity.[1] Prior to use, KOH electrolytes were filtered through a column filled with Chelex® 100 cation exchange resin (Sigma-Aldrich, 50 – 100 mesh) to remove all metal impurities. All solutions were prepared using ultrapure water. The contact angle of the electrolytes were measured by taking an image of the 10 µL droplet on a platinum surface using a custom-made goniometry device.[2]

Electrode preparation

A Pt thin film sputtered on a Si wafer with nanoscale grain boundaries was used to investigate the wetting behavior of alkaline electrolytes. For mapping the facet dependent OER activities, a Pt plate electrode with micrometer scale grain sizes was prepared. First, the Pt plate (Rudolf Flume GmbH) was polished 3 times with 3M lapping papers (3 µm, 1 µm, and 0.3 µm) with ethanol as a lubricant. It was sonicated in ethanol to remove leftovers of the polishing grains between each polishing. Then, it was annealed at 1000 °C for 40 min to increase the grain sizes.[3] The electrode was flame-annealed under a butane flame and cooled at room temperature right before the scanning electrochemical cell microscopy (SECCM) experiment.

SECCM tip preparation

Single barrel quartz capillaries with filaments (1.2 mm outer diameter, 0.9 mm inner diameter, 100 mm length, Sutter Instruments) were pulled using a laser puller (P-2000, Sutter Instruments) with the following pulling parameters: Heat 650, Filament 3, Velocity 30, Delay 130, Pull 85, which provided pipettes with diameters ~ 900 nm. The diameter of the capillaries was determined by SEM imaging (FEI, Quanta 3D ESEM) (Fig. S1). The outer wall of the capillaries was silanized by immersing them in undiluted $SiCl_2(CH_3)_2$. An Ar flow was provided inside the capillaries to prevent the solution from entering inside the tip. The capillaries were kept in the solution for 30 s, then removed and held vertically for an additional 60 s. For the SECCM measurements, silanized pipettes were filled with a KOH-based electrolyte using a Microfil needle with 0.1 mm inner diameter (MF34G-5, World Precision Instruments). For the electrochemical measurements a Ag/AgCl (3 M KCl) was used as a reference-counter electrode (RCE), as depicted in Fig. 1A.

SECCM measurements

All electrochemical measurements were conducted utilizing a home built SECCM setup,[4] employing a single-barrel capillary filled with the electrolyte solution and fixed to a pipette holder. The electrode was placed on top a x, y, z-piezo cube (P-611.3S nanocube, Physik Instrumente) for precise positioning through an analog amplifier (E-664, Physik Instrumente). The area of interest of the Pt substrate was coarsely positioned approximately 30 –40 µm below the SECCM tip by adjusting its coordinates with three stepper motors (Owis) with an L Step PCIe (Lang) controller, coupled with an optical camera (DMK 21AU04, The Imaging Source). The SECCM probe was gently brought in contact with the Pt surface, employing a surface current (i_{surf}) threshold of around 2 pA to detect the moment the meniscus-surface connection was established, halting further translation. Multiple points within this selected area were probed using a standard hopping mode protocol. The entire setup was placed in a Faraday cage equipped with thermal isolation panels (Vaku-Isotherm), on a vibration-dampening table (RS 2000, Newport), supported by four S-2000 stabilizers (Newport) to isolate it from building vibrations. The

system was a two-electrode configuration (Fig. 1A), therefore the current passing through the combined reference/counter electrode (RCE) was quantified using a variable gain transimpedance amplifier (DLPCA-200, FEMTO Messtechnik) to measure i_{surf}. The potential of the Ag/AgCl (3 M KCl) was measured before and after each SECCM experiment, and it did not shift after the SECCM experiments. Data acquisition and instrument operation were managed through an FPGA card (PCIe-7852R), controlled by a modified version of the Warwick Electrochemical Scanning Probe Microscopy software (WEC-SPM) provided by the University of Warwick, developed in LabVIEW (National Instruments). Data processing and analysis were made using the Matlab R2022a (Mathworks) software package. For the reproducible investigation, pipettes with a tip opening diameter of approximately 900 nm were utilized. After conducting the SECCM experiment, the size of the landing spots was measured from the SEM images using ImageJ software. The surface topography of the SECCM footprints after the scan-hopping experiment was evaluated by means of AFM in air (JPK, NanoWizard 3) using a conducting-Si probe coated with 5 nm Cr and 25 nm Pt (ElectriMulti75-G, BudgetSensors).

EBSD characterization

EBSD was performed with a JSM-7200F SEM (JEOL) with an EBSD detector (AZtecSymmetry CMOS detector, Oxford). During the EBSD mapping, the diffraction patterns were acquired every 80 nm with an accelerating voltage of 15 kV on the sample tilted to 70°. The EBSD data was analyzed by the MTEX toolbox (https://mtex-toolbox.github.io/index, accessed March, 2024).[5]

FEM simulation of the SECCM voltammograms

Time-dependent FEM simulations were carried out using COMSOL Multiphysics version 6.2 with the electroanalysis physics module. The pipette body and the droplet were modeled in a 2D axisymmetric cylindrical geometry (Fig. S4). To minimize the computational load, only the last 1.5 mm region of the SECCM tip was considered. This height is much larger than the diffusion layer thickness (approximately 30 μm according to the simulation result), making the simulation outcome unaffected by it. The SECCM electrochemical cell consists of a pipette body and a quarter-ellipse shaped droplet. The parameters defining the tip-droplet geometry were as following: q (semi-angle of the pipette), d_{tip} (the tip opening diameter), d_{drp} (the droplet diameter), h (the thickness of the droplet), and y (the distance between the pipette end and the electrode surface), as illustrated in Fig. S4. The size of meshes was sufficiently dense to depict the nano-to-micro scale concentration distribution of the redox species. (Maximum 5 nm size at the droplet, 5 nm–500 nm mesh size in the pipette body, and maximum 15 μm mesh size in the pipette bulk). The boundary conditions are summarized in Table S1. The mass transport of redox species was calculated by solving the diffusion equation (Eq. S1).

$$\frac{\partial C_i}{\partial t} = - D_i \nabla C_i \tag{S1}$$

In Eq. S1, C_i is the concentration of the species i, t is time, and D_i is the diffusion coefficient of the species i. Migration was not considered due to the high concentration of supporting electrolytes in the experiment. Butler-Volmer kinetics was used to express the potential-dependent flux of the redox species at the electrode-droplet interface (Eq. S2).

$$i = FAk^0[C_R e^{-\frac{\alpha F}{RT}(E - E^0)} - C_O e^{\frac{(1-\alpha)F}{RT}(E - E^0)}] \tag{S2}$$

In Eq. S2, i is the current, F is the Faraday constant, A is the electrode area, k^0 is the standard rate constant, which is 1 cm/s, α is the electron transfer coefficient, which is 0.5, E is the electrode potential, and E^0 is the standard electrode potential (0.23 V for $Fc(MeOH)_2$).

Supplementary Figures

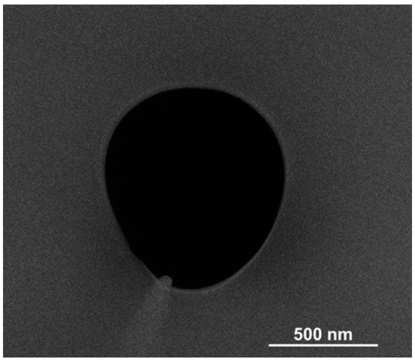

Fig. S1 Representative SEM image of a pipette opening with a diameter of about 900 nm.

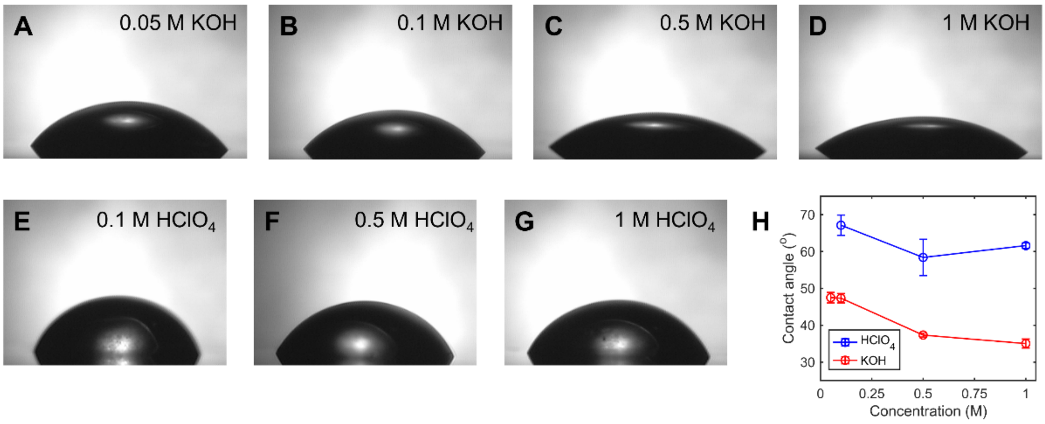

Fig. S2 Representative images of 10 µL droplets of (A–D) KOH solutions and (E–G) $HClO_4$ solutions on a platinum surface. (H) The contact angle of KOH and $HClO_4$ solutions as a function of the electrolyte concentration.

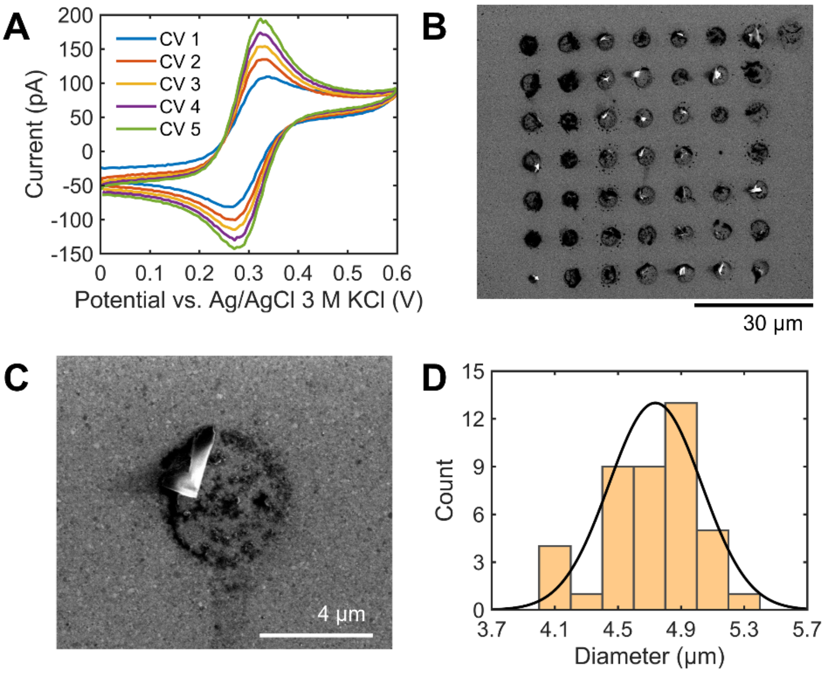

Fig. S3 (A) Sequence of 5 CVs at 1 V/s for 1 M KOH + 1 mM $K_3[Fe(CN)_6]$ recorded at a Pt surface on a single spot after tip landing. (B) SEM image of the 49 landing spots. (C) SEM zoomed image of a single spot showing crystallized KOH on the Pt surface. (D) The size distribution of the 42 landing spots with a normal distribution fit (4.7 ± 0.3 µm).

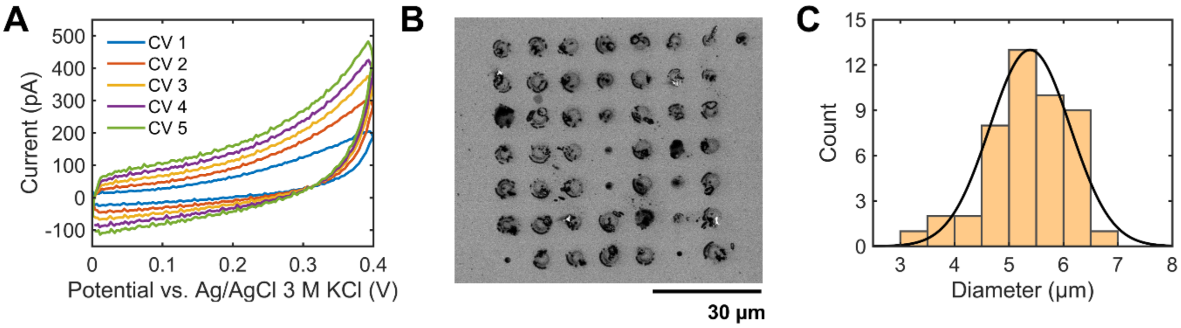

Fig. S4 (A) Serie of 5 CVs at 1V/s for 1 M KOH recorded at a Pt surface in a single spot after tip landing. (B) SEM image of the 49 landing spots. (C) Size distribution of the 46 landing spots with average and standard deviation of 5.4 ± 0.8 μm, respectively.

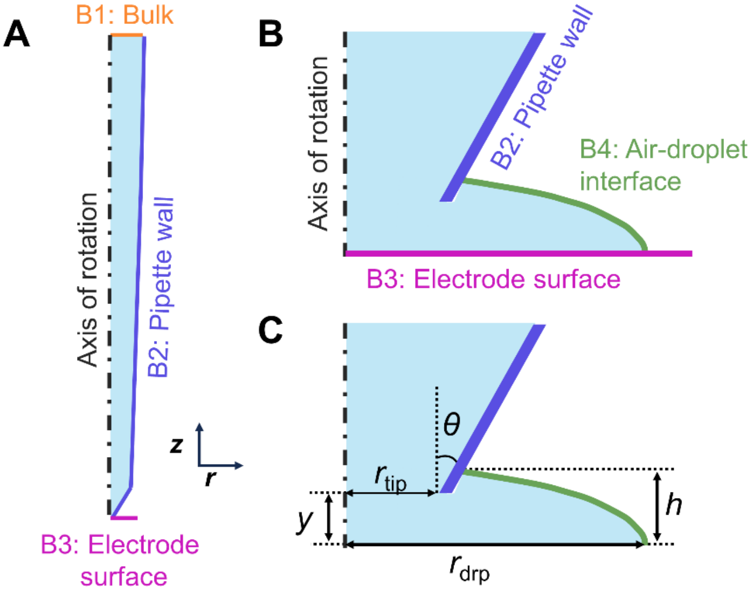

Fig. S5 (A–B) 2D axisymmetric geometry of the SECCM electrochemical cell with "pancake-like" droplet used for the finite element (FEM) simulation. B1, B2, B3 are borders for the material flux, describing the boundary conditions (table S1). (C) The geometry was parameterized as following: q (semi-angle of the pipette), r_{tip} (the tip opening radius), r_{drp} (the droplet radius), h (the thickness of the droplet), and y (the distance between the pipette end and the electrode surface).

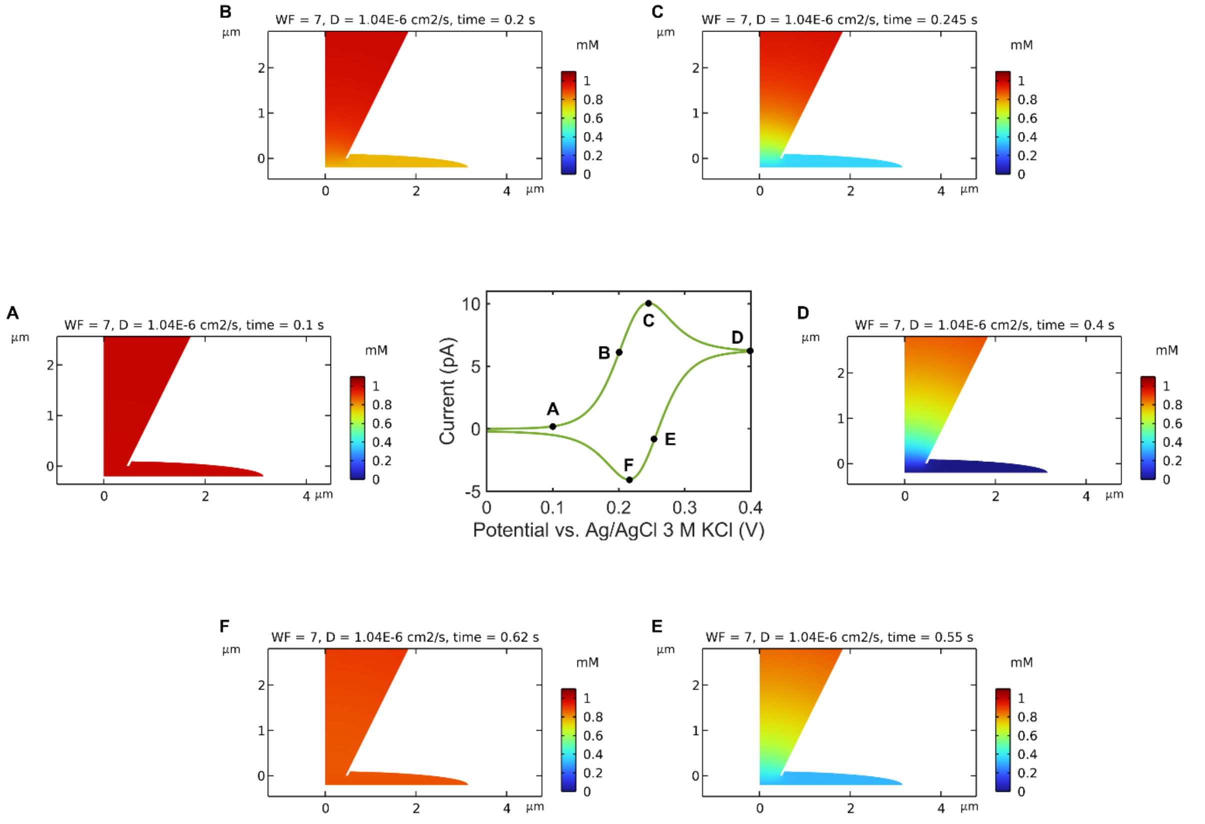

Fig. S6 Simulated SECCM voltammogram for 1 mM $Fc(MeOH)_2$ at 1 V/s and the concentration profile of $Fc(MeOH)_2$ during voltammogram.

As the current increases from A to C, the reactant inside the droplet is mainly consumed, causing a peak-shaped voltammogram which is the characteristic of the thin layer cell mass transport. At D, the reactant inside the droplet is fully depleted and the diffusion layer is formed in the SECCM tip governs the mass transport. This leads to the sigmoidal shaped voltammogram which is the result of the spherical diffusion along the tip.

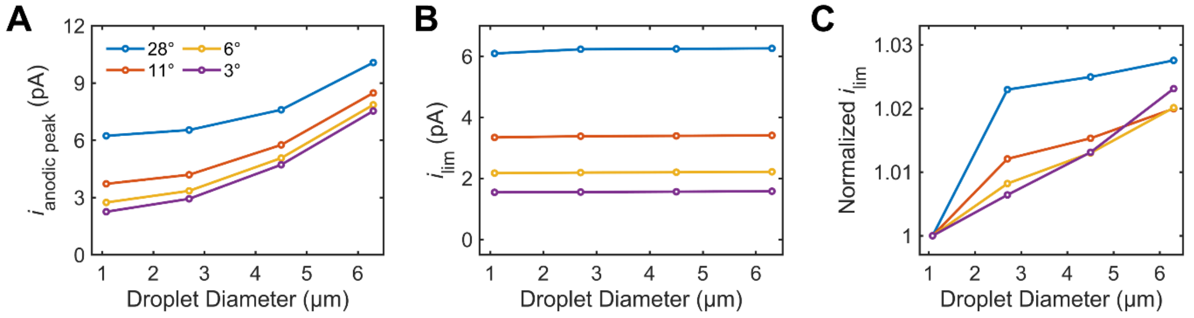

Fig. S7 Simulated electrochemical behavior of the SECCM showing (A) the anodic peak current ($i_{anodic\ peak}$), (B) the limiting current (i_{lim}) and (C) the normalized i_{lim} according to the diameter of the spread droplet.

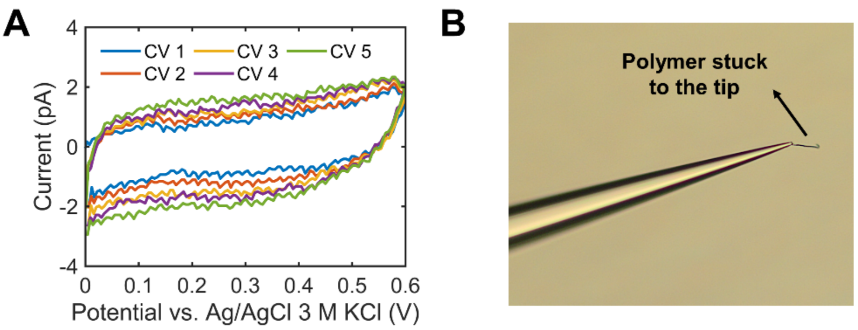

Fig. S8 (A) Serie of 5 CVs at 1V/s for 1 M KOH + 1 mM $K_3[Fe(CN)_6]$ + 0.05% MHEC recorded at a Pt surface in a single spot after tip landing. (B) Polymer protruding from the capillary after the end of the measurement.

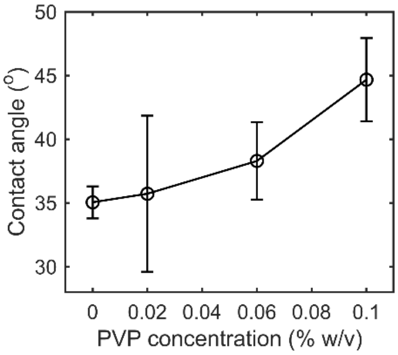

Fig. S9 The contact angle of 1 M KOH solution as a function of PVP concentration.

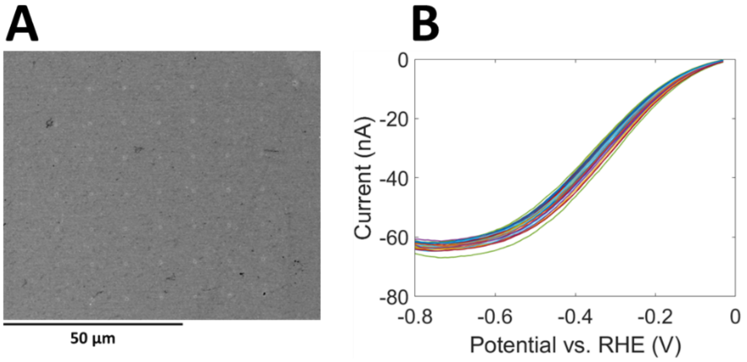

Fig. S10 – *(A) 7 X 7 SEM image showing the landing spots after the SECCM experiments for in 1 M $HClO_4$ + 0.06% PVP using a 180 nm capillary size. (B) Corresponding voltammetric curves from the SECCM mapping in (A).*

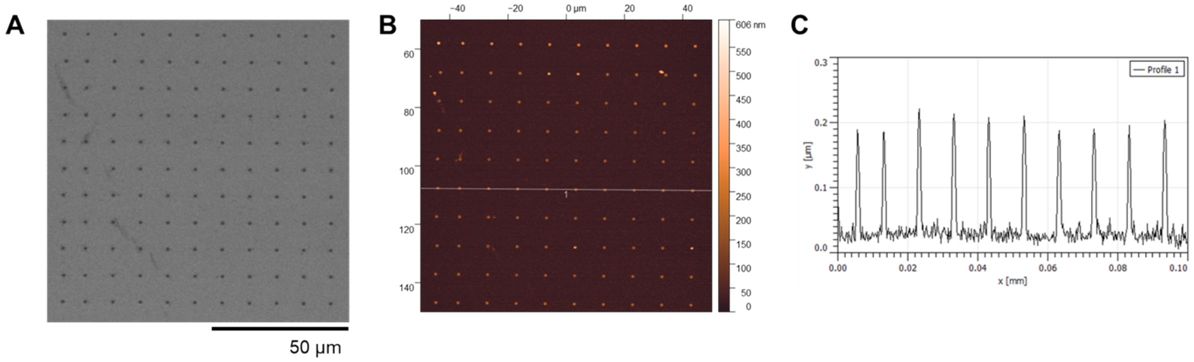

Fig. S11 (A) SEM image after the 11 x 11 SECCM scan-hopping experiment in 1 M KOH + 0.06 % PVP on a polycrystalline Pt surface with nanoscale grains. (B) AFM image of the same SECCM footprints. The white line indicates where the height line profile (C) is measured. (C) The height profile of 11 SECCM footprints.

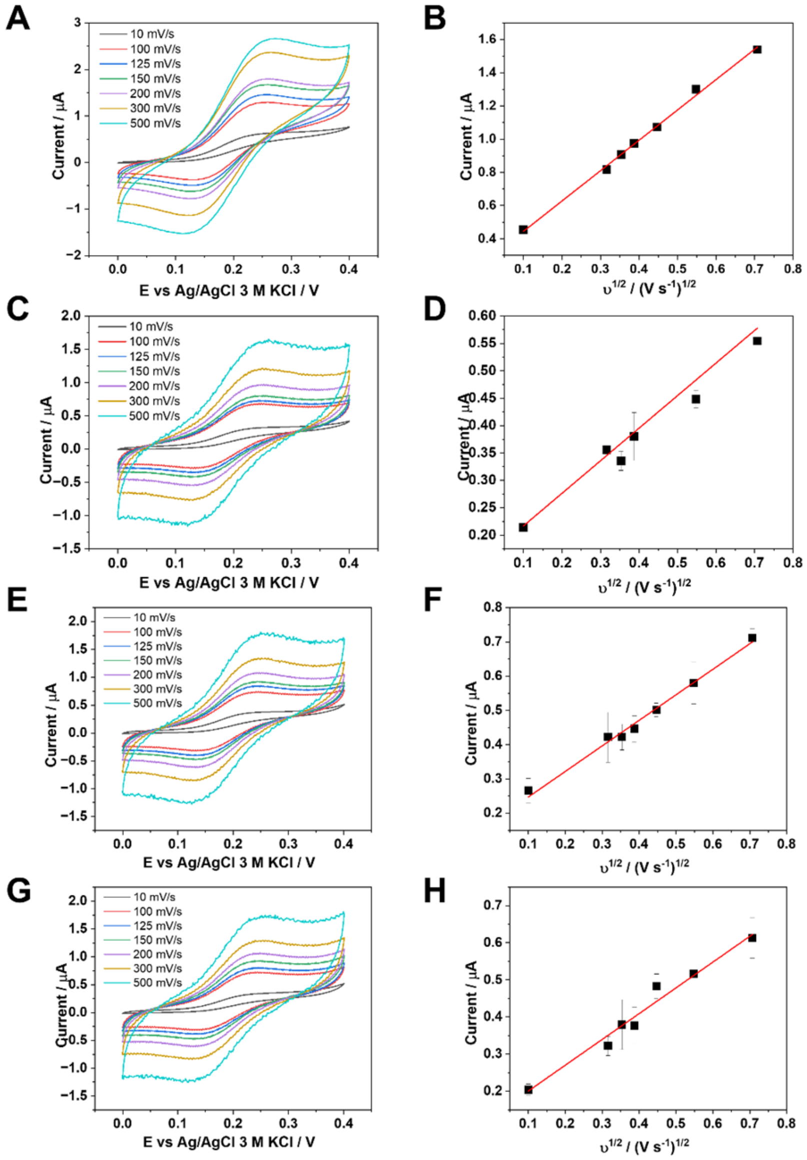

Fig. S12 Series of CVs for PVP + 1 M KOH + 1 mM $Fc(MeOH)_2$ at different scan rates and respective Randles–Ševčík plot for different PVP concentrations: 0 % (A,B), 0.02 % (C,D), 0.06 % (E,F) and 0.1 % (G,H). .

Supplementary Tables

Table S1 Boundary conditions for the COMSOL simulation.

	Boundary	Boundary conditions
**B1**	Bulk reservoir	$C_i = C_{i,bulk}$
**B2**	Pipette wall	$J_i = 0$
**B3**	Air-droplet interface	$J_i = 0$
**B4**	Electrode surface	$J_O = k^0(C_R * e^{-\frac{F}{RT}\alpha(E-E^0)} - C_O * e^{\frac{F}{RT}(1-\alpha)(E-E^0)})$ $J_R = k^0(-C_R * e^{-\frac{F}{RT}\alpha(E-E^0)} + C_O * e^{\frac{F}{RT}(1-\alpha)(E-E^0)})$

Table S2 pH of the electrolytes.

Electrolyte	pH
1 M KOH	13.8
1 M KOH + 0.02 % PVP	13.8
1 M KOH + 0.06 % PVP	13.8
1 M KOH + 0.1 % PVP	13.8

Table S3 Diffusion coefficient (*D*) measured of $Fc(MeOH)_2$ in 1 M KOH with PVP.

**Diffusion coefficient (*D*) of $FcMeOH_2$ (cm^2/s)**	
No polymer	1.04×10^{-6}
0.02 % PVP	1.10×10^{-7}
0.06 % PVP	1.72×10^{-7}
0.1 % PVP	1.50×10^{-7}

References

1 a) W. Li, H. Wang, J. Zhang, Y. Xiang and S. Lu, *ChemSusChem*, 2022, **15**, e202200071; b) J. Swei and J. B. Talbot, *J. Appl. Polym. Sci.*, 2003, **90**, 1153;

2 E. Manderfeld, A. Balasubramaniam, O. Özcan, C. Anderson, J. A. Finlay, A. S. Clare, K. Hunsucker, G. W. Swain and A. Rosenhahn, *Polym. Chem.*, 2023, **14**, 1791.

3 Y. Wang and D. O. Wipf, *J. Electrochem. Soc.*, 2020, **167**, 146502.

4 a) E. B. Tetteh, O. A. Krysiak, A. Savan, M. Kim, R. Zerdoumi, T. D. Chung, A. Ludwig and W. Schuhmann, *Small methods*, 2023, e2301284; b) T. Tarnev, H. B. Aiyappa, A. Botz, T. Erichsen, A. Ernst, C. Andronescu and W. Schuhmann, *Angew. Chem. Int. Ed.*, 2019, **58**, 14265;

5 F. Bachmann, R. Hielscher and H. Schaeben, *SSP*, 2010, **160**, 63.
